# Case report: Squamous cell carcinoma *in situ* of the cornea without involvement of the limbus and conjunctiva

**DOI:** 10.3389/fmed.2024.1418228

**Published:** 2024-11-14

**Authors:** Ji Kyu Yun, Young Joon Ryu, Yongwoo Lee

**Affiliations:** ^1^Department of Ophthalmology, Kangwon National University Hospital, Kangwon National University School of Medicine, Chuncheon, Republic of Korea; ^2^Department of Pathology, Kangwon National University Hospital, Kangwon National University School of Medicine, Chuncheon, Republic of Korea; ^3^Department of Ophthalmology, Columbia University Irving Medical Center, Columbia University, New York, NY, United States

**Keywords:** cornea, ocular surface squamous neoplasia, squamous cell carcinoma, isolated, without limbal involvement

## Abstract

Isolated corneal squamous cell carcinoma without conjunctiva and limbus involvement is rare. We report a case of isolated squamous cell carcinoma *in situ* of the cornea. A 67-year-old male presented with visual disturbance in the left eye (visual acuity, 0.5), with a lesion isolated to the cornea. After an initial diagnosis of keratitis, he was lost to follow-up for 15 months. He subsequently returned for re-evaluation by a corneal specialist owing to progressive visual impairment in the left eye (visual acuity, 0.2). A left scrape biopsy and histopathological examination revealed squamous cell carcinoma *in situ*. No systemic evaluation findings of lymphadenopathy or metastasis were observed. Following removal of the residual mass from the cornea, conjunctival tissue samples, including the limbus, were collected for histopathological examination, with negative findings. No recurrence was observed at 15 months postoperatively. Isolated corneal squamous cell carcinoma *in situ* can be misdiagnosed as simple corneal opacity or keratitis during clinical examination. This condition should be considered in patients with unresponsive keratitis or corneal opacities.

## 1 Introduction

The term ocular surface squamous neoplasia (OSSN), which includes neoplastic lesions arising from the conjunctiva and cornea, was first introduced in 1995 (1). OSSN is of significant clinical concern owing to its resemblance to common conjunctival and corneal surface conditions such as pinguecula, pterygium, conjunctival granulomas, and cysts (2). OSSN encompasses actinic keratosis, dysplasia, carcinoma *in situ*, and invasive squamous cell carcinoma (SCC) (3). Several cases of OSSN involving the limbus or conjunctiva have been reported (4, 5). Most SCCs originate in the limbus, and reports of isolated SCC of the cornea without involvement of the limbus and conjunctiva are extremely rare.

SCC has been reported to occur in >95% of cases involving the limbal area (6). Here, we report a case of isolated corneal SCC, initially misdiagnosed as simple keratitis, without involvement of the limbus or conjunctiva, at initial presentation.

Ethics committee approval to publish this case report was obtained from the Kangwon National University Hospital Institutional Review Board Committee (Reference number: 2023–07–002-001). The patient in this case report provided voluntary informed written consent to the publication of his medical records with anonymized patient information and images.

## 2 Case description

A 67-year-old male employed as a nuclear industry worker complained of a 1-month history of decreased visibility while playing tennis prior to presentation. He was referred to our institution following a local ophthalmic examination with a diagnosis of left keratitis. While he was not directly involved in nuclear reactor operations, he worked in a related capacity. He consumed one glass of makgeolli (traditional Korean rice wine) daily. He had a 30-year smoking history but had quit smoking 20 years ago. He denied any pain or foreign body sensation, visual acuity in his left and right eyes was 0.5 and 0.4, respectively, and intraocular pressure was normal.

### 2.1 Diagnostic assessment

**Figure 1:**
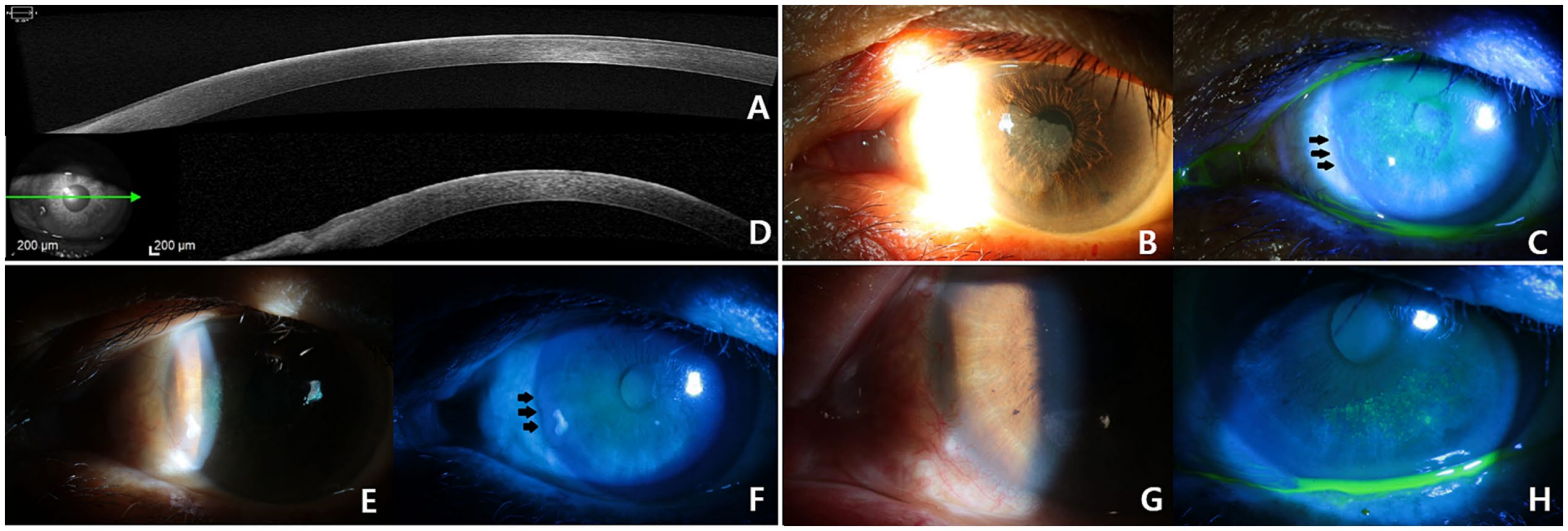
Anterior segment optical coherence tomography (AS-OCT) (A) and corneal infiltration sparing the limbus observed during the initial examination (B,C). The AS-OCT (D) and opaque material sparing the limbus was observed during the follow-up visit after 15 months (E,F). There was no apparent involvement of the corneal limbus or conjunctiva was observed after a left scrape biopsy (G), an opaque material remained (H).

No specific findings were observed during the fundoscopic examination. A rounded and irregular infiltrate with clear borders measuring 5.2 × 6.3 mm in size was observed to be invading the central area of the left cornea. The initial physician prescribed fluorometholone 0.1% (Flumetholon^®^ 0.1 ophthalmic suspension, Santen Pharmaceutical Co., Ltd., Osaka, Japan) and levofloxacin 0.5% (Cravit^®^ ophthalmic solution, Santen Pharmaceutical Co., Ltd., Osaka, Japan) eye drops for the treatment of keratitis and recommended follow-up observation. However, he did not return for follow-up (Figures 1A–C) for 15 months. Owing to worsening visual impairment in the left eye, he sought further evaluation at a local clinic and was subsequently referred to a corneal specialist for further assessment. Visual acuity was 0.2 in the left eye and 0.7 in the right eye, and intraocular pressure was normal. A slit lamp examination revealed an enlarged corneal infiltrate in the left eye. The lesion appeared thicker and darker, with a white gelatinous mass measuring 7.4 × 8.1 mm observed at 8 o’clock. There was no involvement of the limbus or conjunctiva on slit lamp examination.

**Figure 2:**
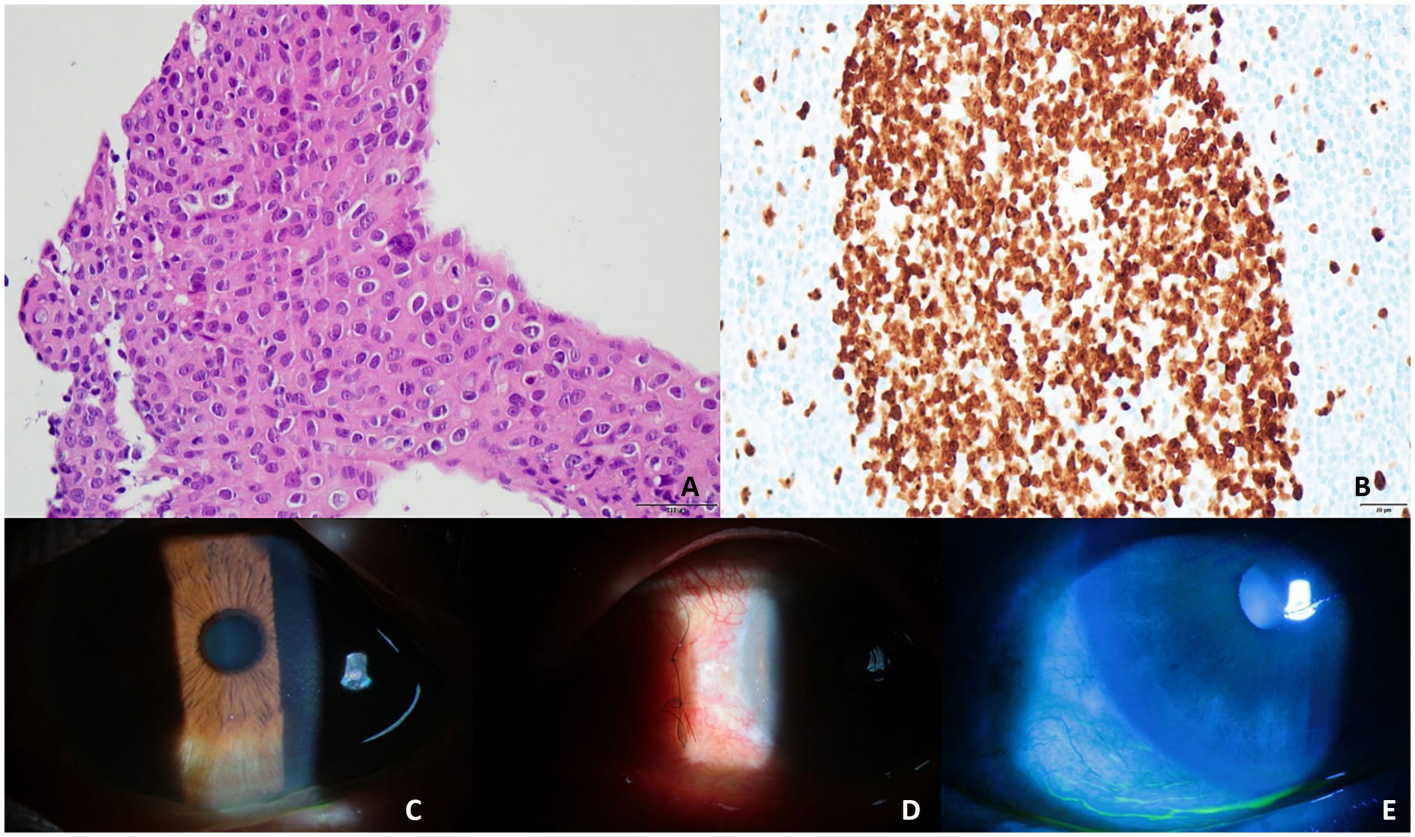
High-magnification images of dysplastic cells showing a high N/C ratio and irregular hyperchromatic nuclei with prominent nucleoli (x400) (A). A uniformly stained pattern throughout the layer can be observed following Ki67 staining (x400) (B). No remnant masses were observed after the additional removal of the left corneal scrape and linear biopsy of the conjunctival margin (C–E).

A left corneal scrape biopsy was performed for diagnostic and treatment purposes (Figures 1D–H). The surgical finding was a whitish patch with a thickened portion in the central area that could not be easily removed. On postoperative day 15, a histopathological examination revealed SCC *in situ* and positive immunostaining for the proliferation marker Ki67. Proliferating dysplastic cells (with enlarged hyperchromatic nuclei and a high nuclear/cytoplasmic ratio) were observed throughout the layer (Figures 2A,B). The patient was immediately referred to our Hematology-Oncology Department for further investigation.

No metastasis was observed on chest radiography, brain magnetic resonance imaging, and computed tomography (CT) scans of the brain, neck, chest, abdomen, and pelvis. No lymph node enlargement, abnormal hypermetabolic lesions, or signs of metastasis were observed on whole-body positron emission tomography (PET)/CT. Postoperatively, the excised portion of the cornea was clean without complications; however, a remnant lesion measuring 3.9 × 2.0 mm remained in the center. Based on the slit lamp examination findings, which indicated no involvement of the limbus or conjunctiva, the remaining corneal epithelial tissue was removed. To further confirm the absence of conjunctival involvement, limbal conjunctival tissue was obtained for histopathological examination.

Mitomycin C (0.02%) soaking was performed for 1 min. After surgery, the remnant mass was completely removed, and the corneal epithelium recovered without complications (Figures 2C–E). Histopathological examination revealed no evidence of limbus or conjunctival involvement. Histopathological examination of the residual mass removed from the cornea revealed SCC *in situ* and Ki67 positivity. After surgery, his visual acuity was 0.7/0.8, and the intraocular pressure was 17/14 mmHg in both eyes. No recurrence was observed at 15 months following the final surgery.

## 3 Discussion

The prevalence of OSSN is generally low. According to epidemiological studies, the incidence of OSSN has been reported to be fewer than 0.2 cases per million individuals per year, based on a survey conducted in the United Kingdom in 1996 (7). However, a study from Australia reported a higher incidence rate, estimating 1.9 cases per 100,000 individuals (8).

Multiple factors influence the development of OSSN. Several studies have linked human papillomavirus, exposure to ultraviolet B light rays, human immunodeficiency viruses (HIV-1 and HIV-2), and hepatitis B and C infections with developing OSSN (9). Other contributing factors include chronic cigarette smoking, the use of petroleum products, having hypopigmented hair and eyes, vitamin A deficiency, and exposure to chemicals such as arsenic and beryllium (10, 11). Additionally, individuals from Britain, Austria, and Switzerland appear to be at higher risk (12).

The choice of treatment may vary depending on the histological results, and it is crucial to diagnose and treat invasive SCC, given its potential for metastasis to other organs.

The risk factors in this case included possible exposure to ultraviolet rays because our patient played tennis as a hobby and had a smoking history; however, he had stopped smoking 20 years prior. The relevance of the patient’s employment as a nuclear industry worker is unclear but cannot be ignored. The limbal area, the border between the conjunctiva and cornea, is a site of mitotic activity. Therefore, OSSN typically originates at the limbus and subsequently extends to the adjacent conjunctiva and cornea (1). Erie et al. reported that, of 98 patients with conjunctival and corneal intraepithelial neoplasia, there was corneal involvement in five cases, limbus involvement in 86 cases, conjunctival involvement in five cases, and the limbal area was involved in all 22 patients with invasive SCC. Despite isolated cases involving only the cornea having been previously reported (6), no such cases have been reported in East Asia.

In this case, although a corneal specialist did not conduct the initial evaluation, corneal infiltration sparing the limbal area led to an initial diagnosis of keratitis. However, a definitive diagnosis was delayed owing to the patient’s loss to follow-up. During re-evaluation 15 months later, infiltrative lesions sparing the limbal area were still observed, which made it difficult to strongly suspect SCC. A tissue biopsy was undertaken to establish a diagnosis and initiate treatment, and the results confirmed SCC *in situ*.

After confirming the absence of systemic metastasis, we planned to excise the remaining tissue. The standard treatment involves wide excision of conjunctiva combined with mitomycin C soaking or cryotherapy. However, based on the slit lamp and OCT findings, we determined that conjunctival involvement was unlikely. Therefore, only remaining corneal lesion was excised, and to assess conjunctival involvement, a 1.5 cm linear excision of the conjunctiva, including the limbus, was performed for biopsy. The biopsy results confirmed no conjunctival invasion, and only additional mitomycin C soaking was performed.

In this study, we reported a case of a patient who initially presented with corneal opacity and was misdiagnosed with keratitis, but was later diagnosed with isolated SCC *in situ* of the cornea. Although SCC of the cornea is uncommon, it can be potentially fatal if it metastasizes to other organs. Therefore, when treating a patient presenting with corneal opacity, although the likelihood is low, the possibility of OSSN should always be considered.

## Data Availability

The original contributions presented in the study are included in the article/supplementary material, further inquiries can be directed to the corresponding author.
